# Interdisciplinary surgical management of multiple retained and impacted teeth in a pediatric patient: a case report

**DOI:** 10.1093/jscr/rjag634

**Published:** 2026-07-29

**Authors:** Areli Guadalupe Flores-Carrillo, Carlos Alberto Zarazúa-González, Karin Anette Orozco-Varela, Marsol Ivani Ortiz-Casillas, Rubén Alberto Bayardo-González, Beatríz Verónica Panduro-Espinoza, Juan Ramón Gómez-Sandoval

**Affiliations:** Department of Integral Dental Clinics, University Center of Health Sciences, University of Guadalajara, Specialty in Pediatric Dentistry (CUCS-UdeG), Sierra Mojada 950, Colonia Independencia Oriente, Guadalajara 44340, Jalisco, Mexico; Department of Integral Dental Clinics, University Center of Health Sciences, University of Guadalajara, Specialty in Pediatric Dentistry (CUCS-UdeG), Sierra Mojada 950, Colonia Independencia Oriente, Guadalajara 44340, Jalisco, Mexico; Department of Integral Dental Clinics, University Center of Health Sciences, University of Guadalajara, Specialty in Pediatric Dentistry (CUCS-UdeG), Sierra Mojada 950, Colonia Independencia Oriente, Guadalajara 44340, Jalisco, Mexico; Department of Integral Dental Clinics, University Center of Health Sciences, University of Guadalajara, Specialty in Pediatric Dentistry (CUCS-UdeG), Sierra Mojada 950, Colonia Independencia Oriente, Guadalajara 44340, Jalisco, Mexico; Department of Integral Dental Clinics, University Center of Health Sciences, University of Guadalajara, Specialty in Pediatric Dentistry (CUCS-UdeG), Sierra Mojada 950, Colonia Independencia Oriente, Guadalajara 44340, Jalisco, Mexico; Department of Human Reproduction Clinics Child Growth and Development, University Center of Health Sciences, University of Guadalajara, Guadalajara 44340, Mexico; Department of Integral Dental Clinics, University Center of Health Sciences, University of Guadalajara, Specialty in Pediatric Dentistry (CUCS-UdeG), Sierra Mojada 950, Colonia Independencia Oriente, Guadalajara 44340, Jalisco, Mexico; Institute of Innovation and Applied Dentistry, Department of Integral Dental Clinics, University Center of Health Sciences, University of Guadalajara (CUCS-UdeG), Sierra Mojada 950, Colonia Independencia Oriente, Guadalajara 44340, Jalisco, Mexico; Institute of Dental Research, Department of Comprehensive Dental Clinics, University Center for Health Sciences, University of Guadalajara (CUCS-UdeG), Sierra Mojada 950, Colonia Independencia Oriente, Guadalajara 44340, Jalisco, Mexico

**Keywords:** retained teeth, dental impaction, oral surgery, interdisciplinary management, case report

## Abstract

Eruption disturbances may generate esthetic, functional, and structural complications in pediatric patients. This report describes a 10-year-old male patient presenting delayed dental eruption, multiple retained and impacted permanent teeth, a supernumerary tooth, and a dentigerous cyst in the mandibular region. Clinical evaluation, panoramic radiography, and cone beam computed tomography revealed severe ectopic positions, risk of root resorption, and mechanical obstructions preventing eruption. A comprehensive single-stage surgical approach was performed under general anesthesia, including extraction of teeth without eruptive potential, cyst enucleation, and surgical exposure of teeth eligible for orthodontic traction. Postoperative evolution was favorable. This case highlights the importance of early diagnosis, three-dimensional planning, and interdisciplinary management to optimize outcomes in complex pediatric tooth retention cases.

## Introduction

Dental eruption is a complex biological process that may be altered by local or systemic factors, resulting in disturbances such as tooth retention and impaction. A retained tooth remains embedded beyond its expected eruption time, whereas impaction occurs when mechanical barriers or unfavorable orientation prevent emergence. These conditions may lead to crowding, cystic lesions, or root resorption [1]. Multiple tooth retention in non-syndromic patients has multifactorial etiology associated with eruptive deviations, bone-tooth discrepancies, or pericoronal pathology. Although supernumerary teeth may contribute to impaction, their prevalence is low, and the occurrence of multiple supernumerary teeth without syndromes such as cleidocranial dysplasia or Gardner syndrome is uncommon [2]. Cone beam computed tomography (CBCT) has improved identification and characterization of retained teeth by enabling accurate evaluation of their relationship with adjacent structures and facilitating surgical planning [3]. Management requires interdisciplinary approach involving oral and maxillofacial surgery, orthodontics, and pediatric dentistry to restore function, occlusion, and normal dental arch development. This report describes surgical management of a pediatric patient with multiple retained and impacted teeth [4].

## Case report

A 10-year-old male patient, ASA I, presented with delayed tooth eruption. No relevant medical, hereditary, or systemic history was reported, and no phenotypic traits associated with hyperdontia-related syndromes were observed. Clinical examination revealed mixed dentition with absence of teeth 11, 12, 13, 14, 15, 21, 22, 23, and 25 in the maxilla, as well as absence of teeth 32 and 33 in the mandible. Primary teeth 55, 83, 84, and 85 showed extensive carious lesions (Fig. 1a and b).

**Figure 1:**
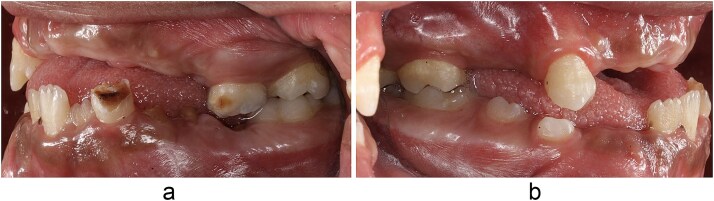
(a) Right intraoral photograph. Multiple missing teeth and carious lesions in primary teeth are observed. (b) Left intraoral photograph. Absence of several permanent teeth is observed, as well as reduced maxillary growth compared with the mandible.

The inconsistent eruption chronology prompted radiographic evaluation. Panoramic radiography revealed multiple retained teeth in both arches (Fig. 2). In the maxilla, teeth 11, 12, 13, 14, 15, 21, 22, 23, and 25 were retained. In the mandible, teeth 32, 33, 43, 44, and 45 were also retained, along with a supernumerary tooth located between teeth 32 and 34. A radiolucent area associated with teeth 44–45 suggested a dentigerous cyst. Severe caries was also observed in tooth 36.

**Figure 2:**
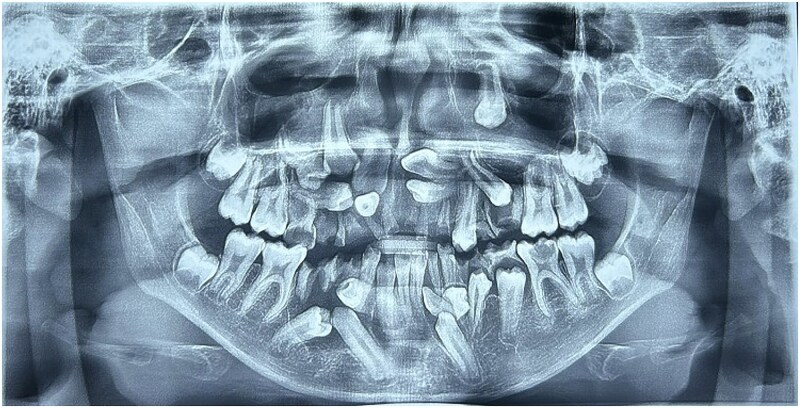
Panoramic radiograph. Retention of multiple permanent teeth is observed, as well as root fragments of primary teeth. A radiolucent area is also noted in the crown of tooth 36 and another radiolucent area in the region of teeth 44 and 45.

CBCT allowed precise determination of the number, position, and anatomical relationships of the involved teeth (Fig. 3). Teeth 13 and 23 showed severe deviation from their normal eruption path, with tooth 23 located atypically near the left infraorbital rim. Teeth 43 and 44 were impacted. Close proximity between impacted maxillary teeth and the roots of teeth 12, 13, and 22 indicated risk of external root resorption. The dentigerous cyst associated with teeth 44–45 was confirmed.

**Figure 3:**
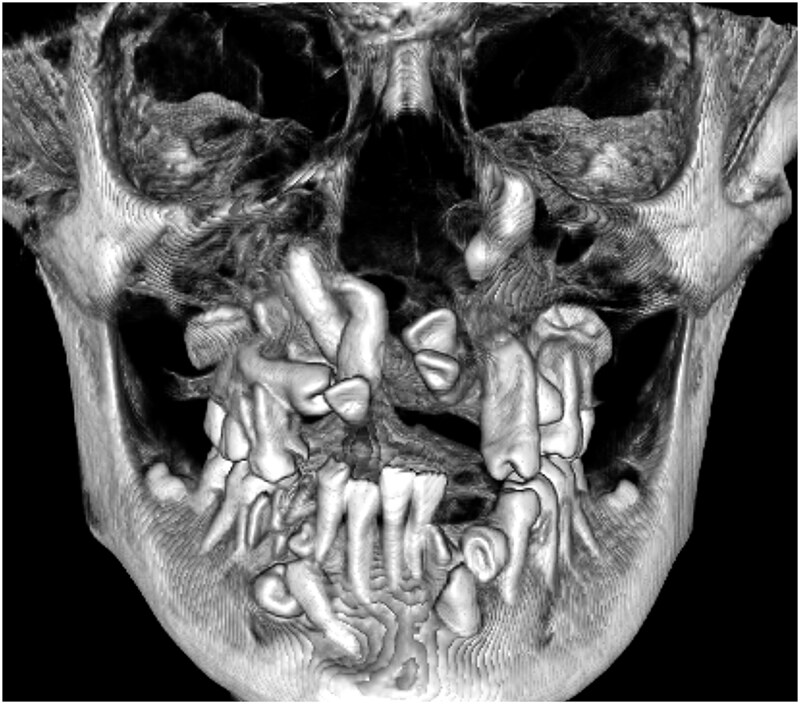
Three-dimensional reconstruction using tomography. Frontal view showing a severe eruptive alteration characterized by multiple retained permanent teeth in ectopic positions, with marked angulations and rotations. Prolonged retention of primary teeth is also observed.

Due to the complexity of the case and the number of involved teeth, the interdisciplinary team (oral and maxillofacial surgery, pediatric dentistry, and orthodontics) decided to perform a comprehensive single-stage surgical approach under general anesthesia. As transoperative analgesia, 2% mepivacaine with epinephrine 1:100 000 was administered, with a total dose of 108 mg (3 cartridges), within the safe dosage range for a 38 kg patient.

In the mandible, primary teeth 83, 84, and 85 were extracted to allow surgical access. A vestibular flap was elevated and conservative osteotomy was performed to remove tooth 44. The lesion associated with teeth 44–45 was enucleated and histopathologically confirmed as a dentigerous cyst. Teeth 33, 43, and 45 were surgically exposed, and orthodontic buttons were placed to facilitate future traction (Fig. 4). Tooth 32 was extracted due to interference with the eruption path, together with a supernumerary tooth in the same region (Fig. 5a and b).

**Figure 4:**
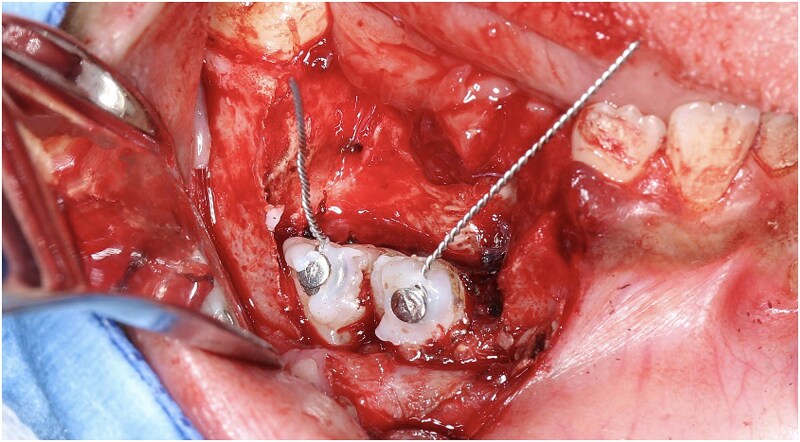
Surgical exposure and placement of orthodontic buttons. Exposure surgery of retained teeth with placement of orthodontic attachments for traction.

**Figure 5:**
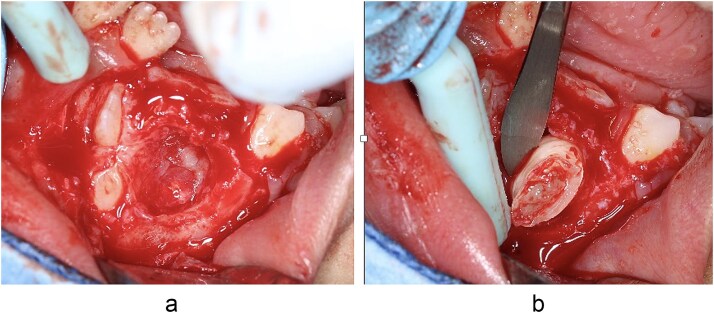
(a) Exposure of retained teeth. Exposure of teeth 32 and 33 is observed following osteotomy. (b) Extraction of a supernumerary tooth. A supernumerary tooth exposed through osteotomy in the lower left quadrant is observed.

In the maxilla, following middle superior alveolar, infraorbital, and nasopalatine nerve blocks, a flap extending from premolar to premolar was elevated. Teeth 11, 12, 13, 21, and 22 were exposed and extracted because their anatomical position made spontaneous eruption unlikely (Fig. 6).

**Figure 6:**
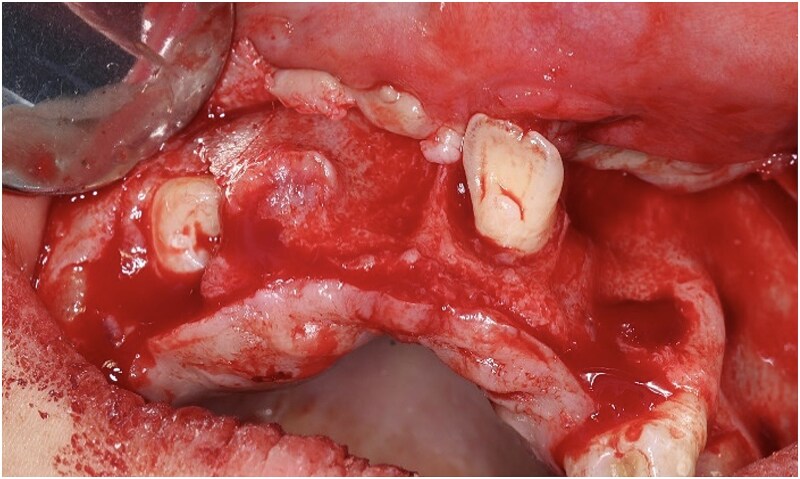
Flap elevation and exposure of maxillary teeth. Exposure of teeth 21, 11, and 12 is observed following flap elevation and osteotomy.

Tooth 23 required a specialized bone window due to its extremely high position (Fig. 7). All flaps were sutured using 3–0 resorbable polyglycolic acid sutures. A total of 14 teeth were extracted, including primary, supernumerary, and retained permanent teeth (Fig. 8).

**Figure 7:**
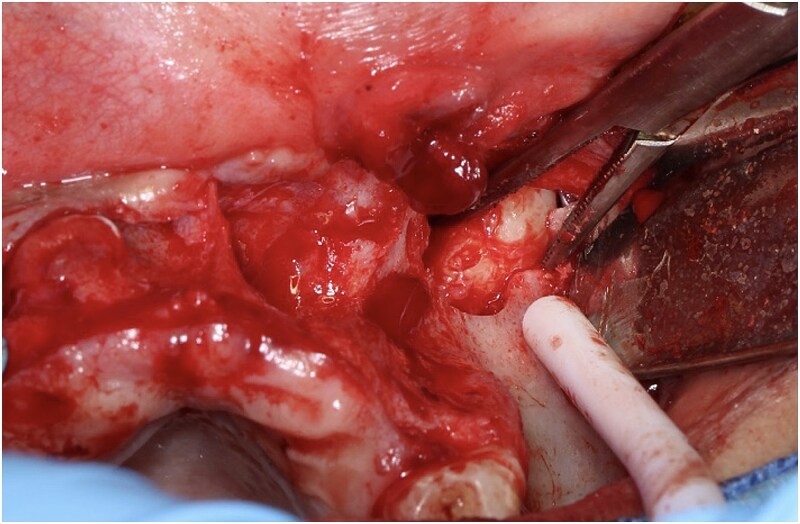
Surgical exposure of tooth 23. Exposure of tooth 23 is observed through a bone window. The tooth was located far from the occlusal plane, near the orbital rim.

**Figure 8:**
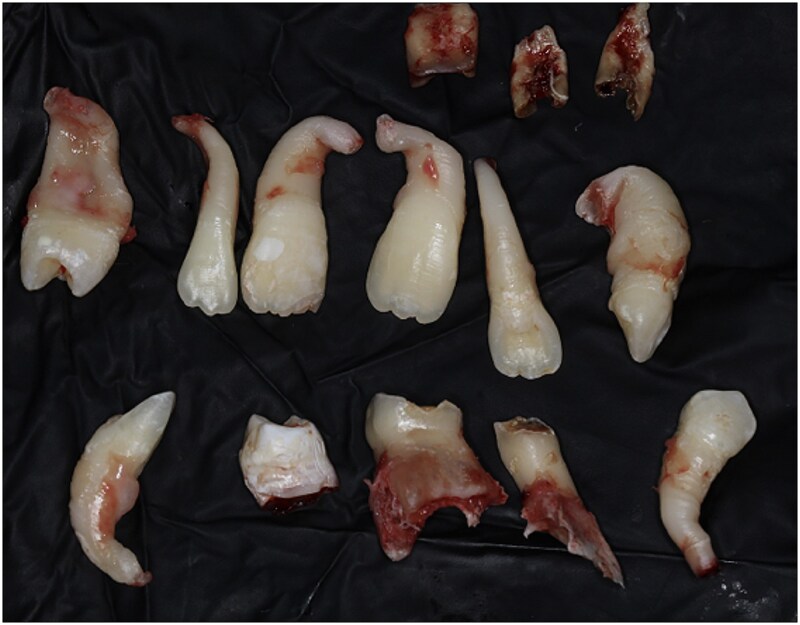
Total number of extracted teeth. A total of 14 teeth were extracted, including primary, permanent, and supernumerary teeth.

Postoperative management included amoxicillin 50 mg/kg/day every 8 hours for 7 days, along with ibuprofen (10 mg/kg) alternated with paracetamol as rescue analgesia. At 7 days, satisfactory healing, mild inflammation, and stability of orthodontic attachments were observed. At 15 days, healing by primary intention was confirmed with no neurological alterations. Orthodontic traction of teeth 33, 43, and 45 was subsequently initiated according to established protocols (Figs. 9 and 10).

**Figure 9:**
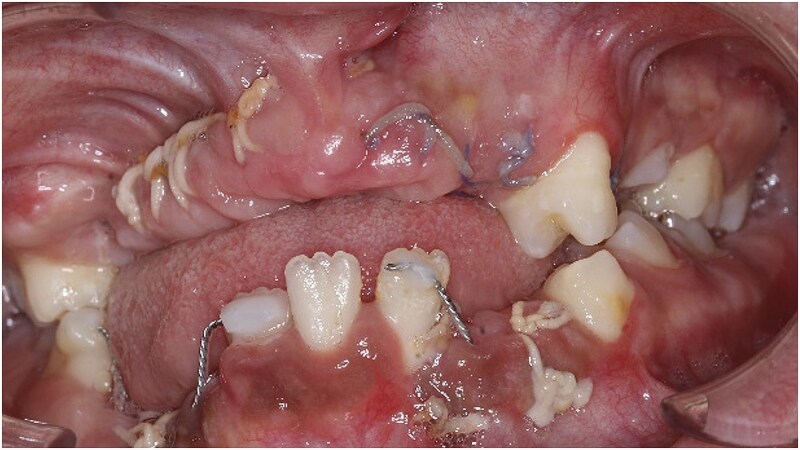
Frontal photograph taken 1 week after the surgical procedure. Presence of 3–0 sutures, food debris retained around the sutures, and mild inflammation in the operated areas are observed, reflecting rapid clinical improvement.

**Figure 10:**
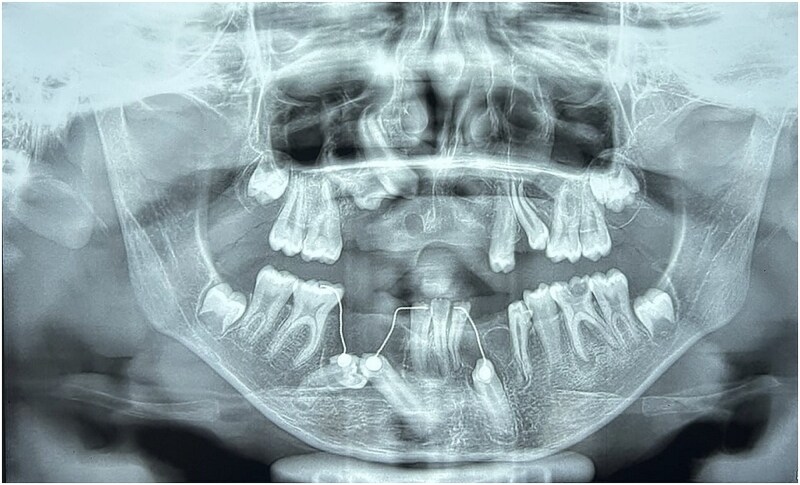
Panoramic radiograph taken 15 days after treatment. Teeth 43, 45, and 33 are observed with orthodontic buttons and ligatures for traction, while teeth 14 and 15 remain retained.

## Discussion

Multiple permanent tooth retention in non-syndromic pediatric patients is uncommon and represents a diagnostic and therapeutic challenge. Its etiology is multifactorial, involving mechanical factors, follicular alterations, bone-tooth discrepancies, and eruptive deviations, as previously reported [5]. In the present case, the coexistence of multiple retained and impacted teeth together with a dentigerous cyst increased the risk of structural and functional complications. These conditions have been associated with root resorption, tooth displacement, and cystic lesion development [6]. Ectopic maxillary canines, such as teeth 13 and 23, also present an increased risk of lateral incisor root resorption when the eruptive trajectory is unfavorable [6], and may occupy atypical anatomical positions requiring specialized surgical approaches [7]. CBCT was decisive in this case, as it improves diagnostic accuracy compared with two-dimensional imaging and allows safer surgical planning [3]. The dentigerous cyst associated with teeth 44–45 was also confirmed, a common complication in cases of prolonged tooth retention [8]. Performing a single-stage surgical approach follows current recommendations aimed at reducing cumulative surgical trauma, minimizing anesthesia exposure, and facilitating early orthodontic management [9]. The literature emphasizes the importance of an interdisciplinary approach to optimize prognosis, particularly in cases involving multiple impactions requiring orthodontic traction and coordinated management among oral surgery, orthodontics, and pediatric dentistry [10]. The favorable clinical outcome observed in this patient supports this treatment strategy.

## Conclusion

The management of multiple retained and impacted teeth in pediatric patients requires comprehensive diagnostic evaluation and an interdisciplinary approach. CBCT is essential for determining the three-dimensional position of impacted teeth and identifying complications such as dentigerous cysts or root resorption. A single-stage surgical approach combined with early orthodontic planning can optimize functional and esthetic outcomes, reduce risks, and facilitate guided eruption of teeth with eruptive potential.

## Data Availability

The data supporting the findings of this study are available from the corresponding author upon reasonable request.

## CARE Checklist (2016) statement

The authors have read the CARE Checklist (2016), and the manuscript was prepared and revised according to the CARE Checklist (2016).
